# Use of hepatic support with MARS in a patient with SARS-CoV-2 Pneumonia, in treatment with ECMO and CRRT therapies: Case Report

**DOI:** 10.1051/ject/2023025

**Published:** 2023-09-08

**Authors:** Irma Villarreal-Ondarza, Cesar Alejandro Rodríguez-Salinas, Rene Gómez-Gutierrez, Israel Guerrero-Izaguirre, Lilia María Rizo-Topete

**Affiliations:** 1 Internal Medicine Resident, Department of Health Sciences, Christus Muguerza Health System, UDEM 64060 Monterrey Nuevo Leon Mexico; 2 Pediatrics, Director of the ECMO Unit, Christus Muguerza Health System, UDEM 64060 Monterrey Nuevo Leon Mexico; 3 Critical Medicine, Internal Medicine, Internal Medicine Professor, Department of Health Sciences, Christus Muguerza Health System, UDEM 64060 Monterrey Nuevo Leon Mexico; 4 Nephrology of the Critically Ill Patient, Internal Medicine, Internal Medicine Professor, Department of Health Sciences, Christus Muguerza Health System, UDEM 64060 Monterrey Nuevo Leon Mexico; 5 Associate Professor of Nephrology Service, Hospital Universitario “José Eleuterio González”, UANL 64460 Monterrey Nuevo Leon Mexico

**Keywords:** Extracorporeal membrane oxygenation, Continuous renal replacement therapy, COVID-19, Acute respiratory distress syndrome, MARS

## Abstract

Extracorporeal Membrane Oxygenation (ECMO) therapy had an important role in the treatment of severe COVID-19 pneumonia, where invasive mechanical ventilation was not enough to provide correct oxygenation to various organ systems. However, there are other extracorporeal technologies, such as the Molecular Absorbent Recirculation System (MARS) and Continuous Renal Replacement Therapy (CRRT), that provide temporal support for any critical patient. The following case describes a 60-year-old man with severe Acute Respiratory Distress Syndrome (ARDS), who needed ECMO therapy. During the critical days of hospitalization, CRRT was used, but a sudden hyperbilirubinemia ensued. Consequently, MARS therapy was initiated; followed by an improvement of bilirubin levels. Additional studies are needed to establish the possible benefits of the combination of MARS therapy and ECMO; however, we detected that concomitantly, there was a decrease in other laboratory parameters such as acute phase reactants. Even though, no change in clinical course was observed, as shown in some studies.

## Overview

The use of Extracorporeal Membrane Oxygenation (ECMO) therapy has increased significantly since the emergence of COVID-19 pneumonia. In critically ill patients with multiple organ failure (MOF), the combination of therapies known as Extracorporeal Organ Support (ECOS) is becoming increasingly common [1, 2] The following describes a patient who required ECOS starting with ECMO, followed by Continuous Renal Replacement Therapy (CRRT), and finally liver support therapy with the Molecular Absorbent Recirculation System (MARS) for severe hyperbilirubinemia. CRRT and MARS were performed while the patient remained on ECMO.

## Clinical case description

This case describes a 60-year-old man with a medical history of systemic arterial hypertension, ischemic heart disease, and hypothyroidism. He was admitted for pneumonia secondary to SARS-CoV-2 with severe Acute Respiratory Distress Syndrome (ARDS), for which elective intubation was decided 2 days after admission; however, in the subsequent 6 h, he did not demonstrate clinical improvement, and venovenous ECMO therapy was initiated. The ECMO cannulation was via a right femoral-internal jugular access, and with a Rotaflow Centrifugal Pump (Maquet/Getinge group; Rastatt, Germany). A 21 Fr jugular cannula and a 27 Fr femoral cannula were used (both from Medtronic; headquarters located in Minneapolis, Minnesota, USA). The ECMO prime consisted of Unfractionated heparin (UFH) at a dose of 10 UI/kg, with a Cardiohelp membrane (Maquet/Getinge group; Rastatt, Germany), starting the system with 3460 revolutions per minute, average flow of 4.6 L/min, FiO2 100% and sweep flow of 6 L/min. Upon initiating ECMO, the labs revealed that the Prothrombin Time (PT) was between 14 and 18 seconds, the Partial Thromboplastin Time (PTT) was between 38 and 75 seconds, INR was between 1.12 and 1.6, and the fibrinogen level was between 274 and 606 (mg/dL).

**Figure 1:**
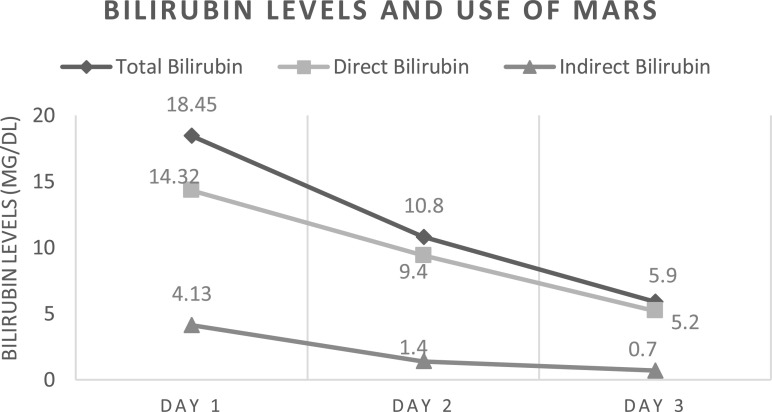
Bilirubin levels and use of MARS.

**Figure 2:**
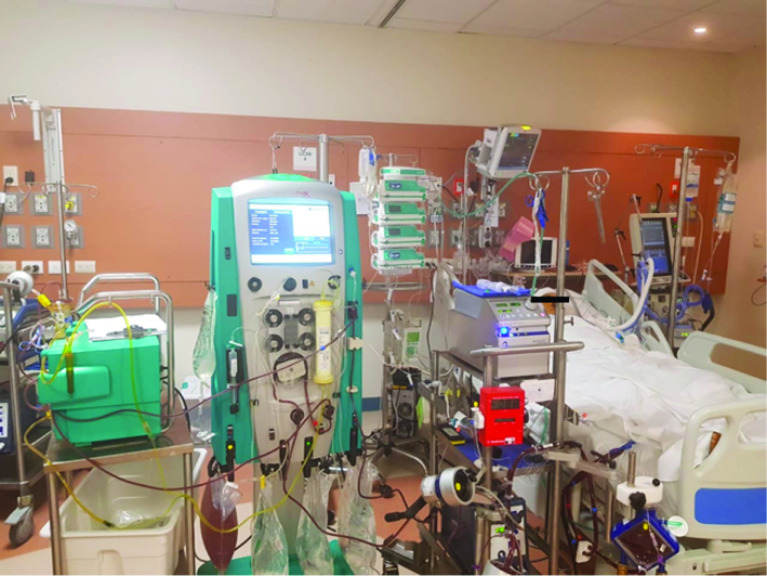
Photo of the actual patient of the clinical case; with the CRRT, ECMO, and MARS functioning.

Twenty days after the start of ECMO, during in-hospital management, he presented with new onsite ischemic cardiovascular complications, manifested with intermittent ventricular tachycardia, requiring angioplasty with the placement of one coronary stent in the Anterior Descending Artery. The patient additionally developed several complications during his course of care including gastrointestinal bleeding due to peptic ulcer, alveolar hemorrhage, and hemothorax. Subsequently, with a long stay, a month after the start of ECMO therapy; he presented septic shock, including a 21,890 K/uL leukocytosis, neutrophils of 18,590 K/uL, fever, neurological alteration, creatinine elevation of 1.46 mg/dL, AST 36 U/L and ALT 27 U/L; increasing 2 days later the AST to 315 U/L and ALT to 145 U/L. Also, during this time period, acute kidney injury (AKI) occurred; with anuria, fluid overload, and hyperkalemia, which precipitated the initiation of CRRT. CRRT was prescribed in venovenous hemodiafiltration modality, the dialysis dose was 30 mL/kg/h with Prismaflex equipment and an Oxiris membrane (both equipment from Gambro, now Baxter; headquarters located in Deerfield, Illinois, USA); no anticoagulation was a need, as the CRRT was connected to the ECMO circuit and the systemic anticoagulation with heparin for ECMO was adequate. The patient then developed hyperbilirubinemia and encephalopathy. As a result, the team decided to initiate liver support which was performed for two, 12-h sessions. The indications were 500 mL 20% human albumin solution, with Blood flow (Qb) and Albumin flow (Qa) at 200 mL/min, and the rest of the prescription was the same as CRRT. With these two sessions, a decrease in bilirubin levels (Figure 1) and an improvement in neurological state were obtained. The MARS sessions were made with a Prismaflex MARS connected to the CRRT Prismaflex machine. Also, during these sessions (MARS and CRRT), other lab values, such as C-reactive protein, decreased from 166 mg/L before these sessions, to a minimum of 50 mg/L days later (Table 1). During the hospital stay, the patient developed several infections, including Klebsiella pneumoniae in the blood culture and Candida auris in the aerobic blood culture drawn from the ECMO and Power-PICC catheter. Multiple antibiotics were used such as ceftaroline, imipenem, linezolid, trimethoprim/sulfamethoxazole, anidulafungin, meropenem, ceftazidime, liposomal amphotericin B, and levofloxacin. Also, the ECMO cannulas were replaced completely, including the ECMO circuit; placing the cannulas in the same position as the previous ones. After the septic shock, there was an improvement in ARDS, allowing a decrease in ECMO support parameters, and lowering the ECMO FiO2 to between 40 and 50%, with a sweep of between 2 and 4 L/min. However, ECMO weaning was never achieved. Despite all treatments and the improvement of hyperbilirubinemia, 2 months later, the patient passed away. Figure 2 shows a picture of the actual patient, with the extracorporeal support described previously.

**Table 1:** Relevant laboratory results according to the beginning of use of CRRT and MARS.

Laboratory results														
Month	April				May									
Day	27	28*	29	30**	1	2	3	4	5	6	7	8	9	10
AST (U/L)	56	87	155	105	127	63	71	54	37	36	43	315	126	124
ALT (U/L)	41	–	56	45	59	53	46	41	30	27	30	145	105	104
ALP (U/L)	103	127	127	148	158	143	142	128	104	100	116	423	313	270
TB (mg/dL)	2.99	7.79	18.45	19.51	10.83	5.97	4.90	3.81	3.44	2.63	2.65	6.26	4.33	3.80
DB (mg/dL)	2.37	5.7	14.32	16.23	9.40	5.22	4.55	3.50	2.94	2.18	2.17	5.38	3.69	3.10
IB (mg/dL)	0.6	2.09	4.13	3.28	1.43	0.73	0.35	0.31	0.50	0.45	0.48	0.88	0.69	0.71
Cr (mg/dL)	0.79	1.25	1.93	2.07	1.57	1.21	1.24	1.12	1.23	1.16	1.14	1.46	1.33	1.13
CRP (mg/L)	137	166	158	–	84	53	–	53	43	–	50	70	79	81
Leu (K/μL)	6.23	5.52	5.52	10.49	10.88	17.71	15.62	15.48	19.10	21.89	17.70	18.82	15.29	12.70
Neu (K/μL)	5.03	4.22	4.22	7.69	11.22	11.22	–	11.89	15.33	18.59	15.05	15.87	12.79	10.40

* CRRT therapy begins.

** MARS therapy begins.

AST: Aspartate aminotransferase, ALT: Alanine transaminase, ALP: Alkaline phosphatase, TB: Total bilirubin, DB: Direct bilirubin, IB: Indirect bilirubin, Cr: Creatinine, CRP: C-reactive protein, Leu: Leukocyte, Neu: Neutrophil, –: no information available.

## Comments

The use of ECMO is reserved as one method of extracorporeal circulation, in cases where gas exchange mechanics is compromised [1]. Another important fact is that in our patient, in addition to ECMO, (due to MOF) other support therapies were used, such as CRRT and the molecular adsorbent recirculation system (MARS^®^, Gambro, Stockholm, Sweden); which consists of an extrahepatic clearance system in patients with severe liver failure who presented hyperbilirubinemia, as well as being useful for the elimination of albumin-bound toxins and soluble toxins. The MARS circuit consists of, an albumin hemodialyzer (MARS flux), a standard high flux hemodialyzer (CRRT), an activated carbon adsorber, and an anion exchanger column [2, 3].

Currently, indications for the use of this therapy are limited to the presence of acute or chronic decompensated liver failure, post-transplant liver dysfunction, multi-organ failure, and persistent pruritus due to cholestasis. A literature search for articles relating to the concurrent use of MARS therapy and ECMO revealed only a few published articles. In our patient, multisystem organ failure with bilirubin elevation was observed, which improved with the use of MARS therapy. However, until this day, the use of MARS therapy is poorly documented in patients with multiple organ failure. One study reviewed several cases of patients with bilirubin levels greater than 17 mg/dL (300 micromoles/liter) that were candidates for MARS therapy, where many of them did not survive; even, all patients with levels greater than 23 mg/dL (400 micromoles/liter) die; considering that normal levels of total bilirubin are from 0.2 to 1.2 mg/dL. This implies that MARS didn′t actually make any significant difference in the mortality of these cases [4]. However, in another retrospective study, with the use of MARS in patients with acute liver failure and the presence of hyperbilirubinemia, greater survival was observed in the group that used ECMO plus MARS, without a greater incidence of adverse events, which showed statistical significance [4, 5]. It should be noted that this study does not mention the inclusion of patients with multisystem organ failure. This is relevant because as already mentioned and observed in our patient, despite an improvement in bilirubin reduction, this therapy does not seem to have an effect on mortality in these types of patients [6, 7].

In conclusion, more studies are needed to establish the possible benefits of the combination of MARS therapy and ECMO. The use of MARS therapy in patients with characteristics such as those presented in this case has scarce evidence. We detected that in our patient, MARS therapy decreased bilirubin levels and clinical improvement; concomitantly, there was a decrease in other laboratory parameters such as acute phase reactants. Even though, no change in clinical course was observed, as shown in other studies, this case report demonstrated that there is no difference in the mortality and survival of these patients.

## Data Availability

The research data associated with this article are included in the article.
